# Efficacy of PE-PLIF with a novel ULBD approach for lumbar degeneration diseases: a large-channel endoscopic retrospective study

**DOI:** 10.1186/s13018-024-04755-3

**Published:** 2024-04-29

**Authors:** Yichi Zhou, Chuankun Zhou, Qingbo Li, Lei Cai, Bowen Kou, Weizhi Fang, Zhi Yao, Mengcheng Wei, Shishuang Zhang, Weijun Liu

**Affiliations:** Wuhan Fourth Hospital, Wuhan, China

**Keywords:** PE-PLIF, ULBD, Large-channel endoscope, Non-touch over-Top technique

## Abstract

**Purpose:**

This study aims to assess the effectiveness of Percutaneous Endoscopic Posterior Lumbar Interbody Fusion (PE-PLIF) combined with a novel Unilateral Laminotomy for Bilateral Decompression (ULBD) approach using a large-channel endoscope in treating Lumbar Degenerative Diseases (LDD).

**Methods:**

This retrospective analysis evaluates 41 LDD patients treated with PE-PLIF and ULBD from January 2021 to June 2023. A novel ULBD approach, called ‘Non-touch Over-Top’ technique, was utilized in this study. We compared preoperative and postoperative metrics such as demographic data, Visual Analogue Scale (VAS) for pain, Oswestry Disability Index (ODI), Japanese Orthopedic Association (JOA) score, surgical details, and radiographic changes.

**Results:**

The average follow-up duration was 14.41 ± 2.86 months. Notable improvements were observed postoperatively in VAS scores for back and leg pain (from 5.56 ± 0.20 and 6.95 ± 0.24 to 0.20 ± 0.06 and 0.12 ± 0.05), ODI (from 58.68 ± 0.80% to 8.10 ± 0.49%), and JOA scores (from 9.37 ± 0.37 to 25.07 ± 0.38). Radiographic measurements showed significant improvements in lumbar and segmental lordosis angles, disc height, and spinal canal area. A high fusion rate (97.56% at 6 months, 100% at 12 months) and a low cage subsidence rate (2.44%) were noted.

**Conclusions:**

PE-PLIF combined with the novel ULBD technique via a large-channel endoscope offers significant short-term benefits for LDD management. The procedure effectively expands spinal canal volume, decompresses nerve structures, improves lumbar alignment, and stabilizes the spine. Notably, it improves patients' quality of life and minimizes complications, highlighting its potential as a promising LDD treatment option.

**Supplementary Information:**

The online version contains supplementary material available at 10.1186/s13018-024-04755-3.

## Introduction

Lumbar degenerative diseases (LDD) include lumbar disc herniation (LDH), lumbar spinal stenosis (LSS), lumbar spondylolisthesis, which commonly present with lower back pain, leg pain, and motor dysfunction, and can severely impact patients' quality of life [1]. In patients with severe symptoms unresponsive to conservative management, surgery has been shown to effectively and rapidly improve clinical symptoms and enhance radiographic parameters of the lumbar spine [2, 3]. However, traditional open lumbar fusion surgeries are associated with greater risk of complications such as blood loss, infection, longer hospital stays, and higher cost [4], making it a challenge to achieve satisfactory clinical outcomes in the treatment of lumbar degenerative diseases [5]. Therefore, exploring a safe and effective surgical approach has become a focal point in the field of spinal surgery.

In recent years, with the continuous development of spinal endoscopic technology, percutaneous endoscopic posterior lumbar interbody fusion (PE-PLIF) has gradually entered the horizon [6]. At the same time, ULBD technology can decompress both sides of the spinal canal through a single incision. It has been reported that the combination of percutaneous endoscopic interlaminar decompression (PEID) and ULBD technology has achieved favorable clinical results in the treatment of lumbar spinal stenosis (LSS) [7–9]. However, the ULBD procedure in the above studies was performed using the UBE system or traditional endoscope with an outer diameter (OD) of 7.2 mm, while the specific technical note on the use of the large endoscope has not yet been reported. Therefore, this study aims to investigate the efficacy and safety of large endoscopic PE-PFLIF combined with a novel ULBD technique in the treatment of LDD.

## Materials and methods

### Study design

Ethical approval for this study was granted by the Ethics Committee of Wuhan Fourth Hospital (approval number KY2023-111-01), and informed consent was obtained from all participating patients. The present study was a retrospective study utilizing the guidelines of Strengthening the Reporting of Observational Studies in Epidemiology (STROBE) [10]. All the surgeries were performed by the same spinal surgical team. Forty-one degenerative lumbar spinal stenosis patients underwent single-segmental PE-PLIF combined with ULBD under large-channel at Wuhan Fourth Hospital between January 2021 and June 2023 were enrolled in this study. The inclusion criteria were as follows: (1) patients with single-segmental LSS exhibiting bilateral lower limb symptoms, irrespective of the presence or absence of lumbar spinal instability; and (2) patients with single-segmental LSS exhibiting bilateral lower limb symptoms with or without unilateral lumbar disc herniation; and (3) inefficacy after ≥ 3 months of strictly conservative treatment, or symptoms were progressively aggravated. The exclusion criteria were as follows: (1) multisegment LSS confirmed by imaging examination; or (2) Spinal infection, spinal tuberculosis, and tumor.

## Surgical techniques

All operations were performed under general anesthesia. Patients were placed in a prone position, and the abdomen was suspended using a U-shaped pad to minimize intraoperative bleeding. Four pedicle guide wires were implanted under the guidance of G-arm fluoroscopy, respectively. A longitudinal incision approximately 12 mm in length was made, and the soft tissue was gradually expanded by serial dilators to facilitate insertion of a working sleeve which docked on the middle line of the ipsilateral inferior articular process facet. The iLESSYS DELTA endoscope (joimax, Germany, Table 1) was then inserted in the working sleeve, and the pressure of the pump of constant saline irrigation was set at 90–110 mmHg**.** The decompression procedure of ipsilateral spinal canal, as previously illustrated [11], is briefly described below. The ipsilateral cranial and caudal laminectomy and partial facetectomy was performed by using the visualized trephine and endoscopic Kerrison rongeur, and the attachments of the ipsilateral LF (ligamentum flavum) was then exposed and detached. After identifying the base of the spinous process (BAP), the contralateral laminectomy was performed by the endoscopic high speed drill. The bony structure of contralateral lamina was removed as much as possible. The facet of the contralateral superior articular process (SAP) was considered as an important landmark (Additional file 1: video S1 ). Afterwards, the contralateral LF was carefully removed in piecemeal using the endoscopic Kerrison rongeur. Subsequently, the contralateral LR was decompressed using an endoscopic Kerrison rongeur, and the contralateral traversing nerve root was displayed. The ipsilateral LF was excised in en bloc, and the discectomy was performed through the ipsilateral side. Specifically, the endpoint of decompression was achieving relaxation of the dural sac and nerve roots. The cartilaginous endplate of vertebral was efficiently scraped off by the visualized osteotome. Adequate autogenous and allogenic bone (Shanxi Osteorad Biomaterial corporation, Shanxi, China) were implanted in the intervertebral space, and a height-adjustable metal interbody fusion cage (REACH-MED corporation, Shanghai, China) was inserted into the intervertebral space. Finally, four pedicle screws with proper size were implanted through the guide wires. The position of all internal fixators was confirmed by fluoroscopy. A closed suction drain was implanted, and the incision was sutured with 3–0 silk suture.

**Table 1:** Parameters of surgical instruments

Endoscope				Working sleeve			Endoscopic high speed drill		
WCL(mm)	WCD(mm)	OD(mm)	LA(°)	L(mm)	ID(mm)	OD(mm)	L(mm)	DD(mm)	RS(r/min)
125	6	10	15	125	10.2	11.2	320	3.5	30000

WCL, Working channel length; WCD, Working channel diameter; OD, Outer diameter; LA, Lens angle; L, length; ID, Inner diameter; DD, drill diameters; RS, rotational speed

## Postoperative care

The drainage tube was removed 24 h after operation, then patients were asked to initiate out-of-bed activity with spinal brace. Functional exercise was performed immediately after surgery. Antibiotics, non-steroidal analgesics, and dehydration drugs were administered simultaneously after surgery.

## Clinical data and radiographic parameters assessment

Clinical data such as operative time, ULBD time, intraoperative blood loss, volume of drainage, post-operation hospital stay and complications were recorded. Visual analogue scale (VAS) for back and leg pain, Oswestry Disability Index (ODI) and Japanese Orthopadics Association (JOA) scores were calculated preoperatively and at 1 day, 1 month, 6 months and 12 months after surgery to evaluate the clinical efficacy.

The radiographic parameters, including lumbar lordotic angle (LLA), segmental lordotic angle (SLA), disk height (DH), cross-sectional area of the spinal canal (CSCA), were recorded preoperatively and at 6 months and 12 months after surgery. LLA, SLA and DH was measured by X-ray (DRVM 1.5, Philips, Germany). Cage subsidence, CSCA and fusion rate was evaluated and calculated by 64-row high-resolution CT (Siemens, Germany).

## Statistical analysis

The data of categorical variables were expressed as frequency and percentage. The data of numerical variables were represented by mean and standard deviation. Normality was determined using the Shapiro–Wilk test. Comparison of data between the two groups in this study was performed using repeated ANOVA. Statistically significant difference at* P* < 0.05. Statistical analysis was performed using SPSS 25.0 software (IBM, USA).

## Results

### Basic demographic information

A total of 41 patients with a mean age of 57.51 ± 8.21 years were included in this study, 17 males (41.46%) and 24 females (58.54%). All patients had an average BMI of 22.24 ± 1.74 kg/m^2^. All patients were followed up for 6 ~ 18 months, with an average follow-up time of 14.07 ± 3.45 months. Among the patients, 16 were hypertension, 8 were diagnosed with diabetes mellitus, and 19 were diagnosed with osteoporosis. The distribution of the surgical segments and the diagnosis of the patients is summarized in Table 2.

**Table 2:** Patient demographics and baseline data (n = 41)

	(n/%, mean ± deviation)
*Sex(n/%)*	
Male	17/41.46
Female	24/58.54
Age(years)	57.51 ± 15.21
BMI(kg/m^2^)	22.24 ± 1.74
*Comorbidity(n/%)*	
Hypertension	16/39.02
Osteoporosis	19/46.34
Diabetes mellitus	8/19.51
Follow-up duration(mon)	14.41 ± 2.86
*Surgical segment(n/%)*	
L_4-5_	29/70.73
L_5_S_1_	12/29.27
*Diagnosis(n/%)*	
LSS	13/31.71
LSS + LDH	5/12.20
LSS + Spondylolisthesis	23/56.10

BMI, body mass index; LDH, lumbar disc herniation; LSS, lumbar spinal stenosis

## Surgical data and complications

The operative time was 186.05 ± 12.92 min, of which ULBD time was 51.83 ± 6.44 min, intraoperative blood loss was 78.66 ± 11.50 ml, postoperative drainage was 29.78 ± 12.66 ml, and postoperative hospital stay was 4.66 ± 1.34 days. Postoperative complications included one incisional infection, two postoperative intraspinal hematoma, and one cerebrospinal fluid (CSF) leak. The patient with incisional infection and hematoma underwent debridement immediately after the onset of fever and wound pain, and the symptoms of infection were controlled after the postoperative change of sensitive antibiotics. One patient experienced dural sac tear and CSF leakage with no significant discomfort. After postoperative elevation of the drainage bag and prophylactic antibiotics, the patient's drainage gradually decreased and the drainage tube was removed on the fourth postoperative day. Table 3 shows the surgical data and complications.

**Table 3:** Surgical data and complications (n = 41)

	(n/%, mean ± deviation)
Operative time(min)	186.05 ± 12.92
ULBD time(min)	51.83 ± 6.44
Intraoperative blood loss(ml)	78.66 ± 11.50
Volume of drainage(ml)	29.78 ± 12.66
Post-op hospital stay(days)	4.66 ± 1.34
*Complications (n/%)*	
Nerve root injury	0/0
Dural sac tear	1/2.44
Incision infection	1/2.44
CSF leakage	1/2.44

ULBD, unilateral laminotomy for bilateral decompression; Post-op, post-operation; CSF, cerebrospinal fluid

## Perioperative functional evaluation

The ODI, VAS and JOA scores were used to assess the patients' clinical symptoms at different times and were measured by repeated measures ANOVA. As shown in Table 4, the VAS and ODI scores decreased significantly over time, with a significant difference at the last follow-up compared to the preoperative period (*P* < 0.001). In addition, the JOA score also changed significantly over time (*P* < 0.001). The patient's symptoms were effectively improved.

**Table 4:** Results of functional evaluation (n = 41)

	Pre-op	1 d	1 mon	6 mon	12 mon	*P* value^*^
VAS back pain	5.56 ± 0.20	1.59 ± 0.18	0.78 ± 0.12	0.39 ± 0.09	0.20 ± 0.06	< 0.001
VAS leg pain	6.95 ± 0.24	1.71 ± 0.15	1.00 ± 0.12	0.24 ± 0.07	0.12 ± 0.05	< 0.001
ODI(%)	58.68 ± 0.80	16.34 ± 0.58	13.07 ± 0.48	10.34 ± 0.38	8.10 ± 0.49	< 0.001
JOA scores	9.37 ± 0.37	18.61 ± 0.34	22.49 ± 0.46	24.07 ± 0.41	25.07 ± 0.38	< 0.001

VAS, visual analogue scale; ODI, Oswestry disability index; JOA, Japanese Orthopaedic Association

*The* P* value is the comparison between pre-operation and last follow-up with repeated measures ANOVA

## Radiographic evaluations

X-ray examination showed that post-operative LLA, SLA and DH were significantly increased in comparison with preoperative values. To assess CSAC, each patient underwent CT at 6 months and 12 months postoperatively. The statistical results (Table 5) showed a significant increase in CSAC postoperatively compared to preoperatively, with a continuous increase in CSAC at 6 months and 12 months postoperatively. Table 5 shows the Cage subsidence rate and fusion rates at 6 months and 12 months after surgery.

**Table 5:** Outcomes of radiographs changes

	Pre-op	6 mon	12 mon	*P* value^*^
LLA(°)	34.04 ± 0.25	45.66 ± 0.17	45.56 ± 0.17	< 0.05
SLA(°)	9.50 ± 0.11	20.90 ± 0.11	20.71 ± 0.11	< 0.05
DH(mm)	7.19 ± 0.13	11.89 ± 0.65	11.79 ± 0.69	< 0.05
CSAC(mm^2^)	98.20 ± 2.57	193.88 ± 4.75	200.49 ± 4.73	< 0.05
Cage subsidence (%)	–	2.44	2.44	–

LLA, lumbar lordotic angle; SLA, segmental lordotic angle, DH, disk height, CSAC, cross-sectional area of the spinal canal. *The P value is the comparison between pre-operation and last follow-up with repeated measures ANOVA

## Discussion

As the population ages, the incidence of Lumbar Degenerative Diseases (LDD) is steadily increase [12]. LDD lead to lower back pain, limb numbness, and reduced lower limb muscle strength, significantly impacting patients' quality of life. Moreover, LDD poses a substantial economic burden on both individuals and society [13].

Minimally invasive spinal techniques, especially spinal endoscopy, have become increasingly popular for treating LDD, owing to their significant advantages in surgical outcomes and patient recovery [14–17]. Among these, percutaneous endoscopic lumbar interbody fusion (PE-LIF) is favored for minimal surgical trauma, safety, reduced postoperative pain, less hidden blood loss, and quicker rehabilitation, alongside robust internal fixation [18–24]. Complementing these techniques, the unilateral laminotomy for bilateral decompression (ULBD) facilitates sufficient decompression through a single incision, minimizing spinal muscle damage and contributing to the overall minimally invasive approach [8, 9, 25, 26]. In previous studies, surgeons have utilized the traditional small endoscope for ULBD by directly traversing the epidural space [8, 9, 25–27]. For example, the protective sleeve of the uniportal bilateral endoscopy (UBE) system’s high-speed drill has been employed to compress the LF, facilitating direct decompression of the contralateral LR [28, 29]. Similarly, a traditional 7.2 mm or smaller uniportal endoscope (UE) can achieve contralateral LR decompression by traversing the epidural space [8]. However, in cases of lumbar vertebral instability or spondylolisthesis, interbody fusion becomes a necessary adjunct to ensure stability.

**Fig. 1:**
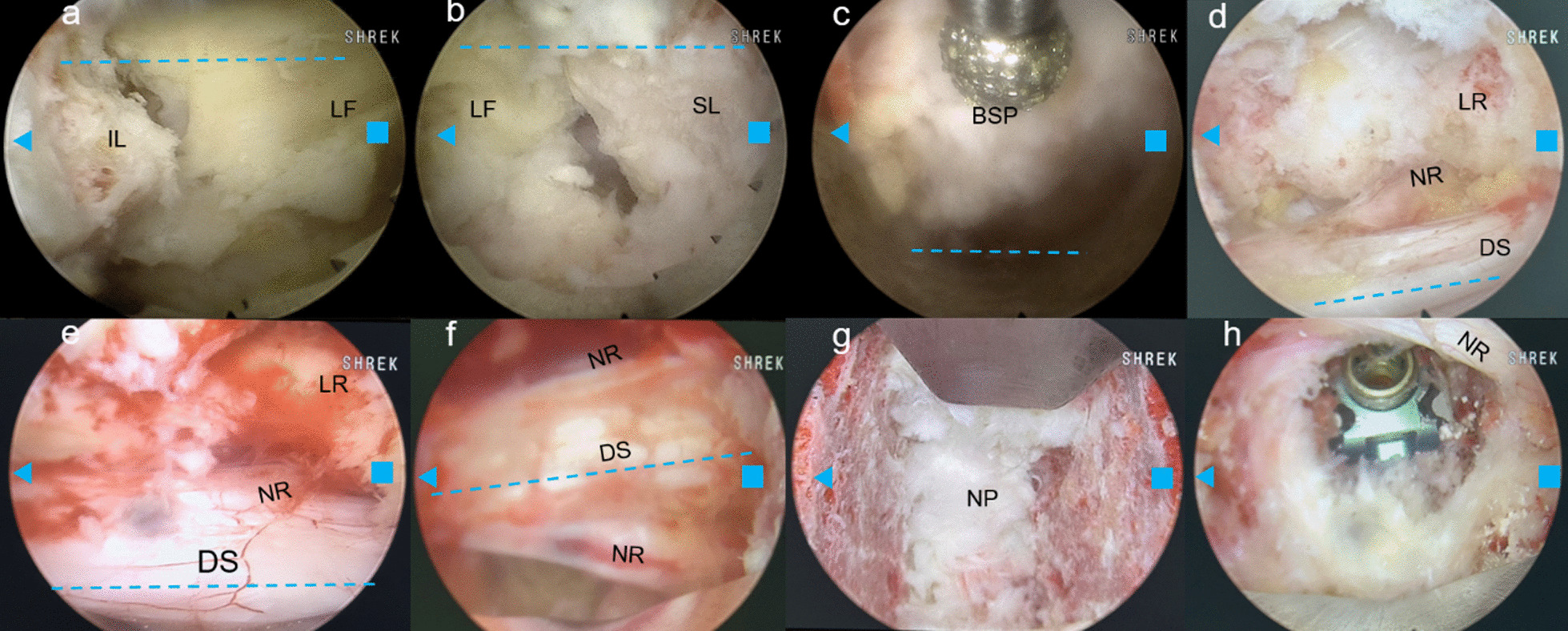
Endoscopic view of the ‘Non-touch Over-Top’ technique. **a**, **b** The interlaminar window was expanded using an endoscopic trepine, and the upper and lower endpoints of the LF were detached. **c** Initially performing ULBD using the endoscopic high-speed drill. **d**,** e** Decompression of contralateral LR. **f** DS and bilateral exiting nerve roots after ULBD. **g** Intervertebral space after discectomy. **h** Intervertebral bone grafting and cage implantation. The blue dashed line represents the median of the spinal canal, the blue triangle represents the cranial side, the blue square represents the cauda side. *ULBD* unilateral laminotomy for bilateral decompression, *LR* lateral recess, *DS* dural sac, *LF* ligamentum flavum, *BSP* base of spinous process, *IL* inferior lamina, *SL* superior lamina, *NR* nerve root, *NP* nucleus pulposus

Traditional UE and UBE systems have been less effective in performing endoscopic interbody fusion procedures due to their narrow field of view and limited Working Channel Diameter (WCD). This raises a critical question: how can we efficiently execute both endoscopic interbody fusion and ULBD in patients with bilateral symptomatic LSS and lumbar spinal instability? The large-channel endoscope, with its wider WCD (Table 1), is adept at performing unilateral endoscopic decompression and fusion. However, its use in ULBD is challenging due to the potential risk of nerve root and dural sac damage when traversing the epidural space, given its large Outer Diameter (Table 1). To date, there have been few studies on performing ULBD with a large endoscope. To address this gap, we have developed the ‘Non-touch Over-Top’ technique. This innovative method allows minimally instruments traversing the space between dural sac and contralateral lamina. It ensures that neither the LF nor the dural sac is compressed by the endoscope or the working sleeve, which does not traverse this space at any point (Fig. 1). The typical six steps of the ‘Non-touch Over-Top’ technique have been illustrated in Fig. 3.

**Fig. 2:**
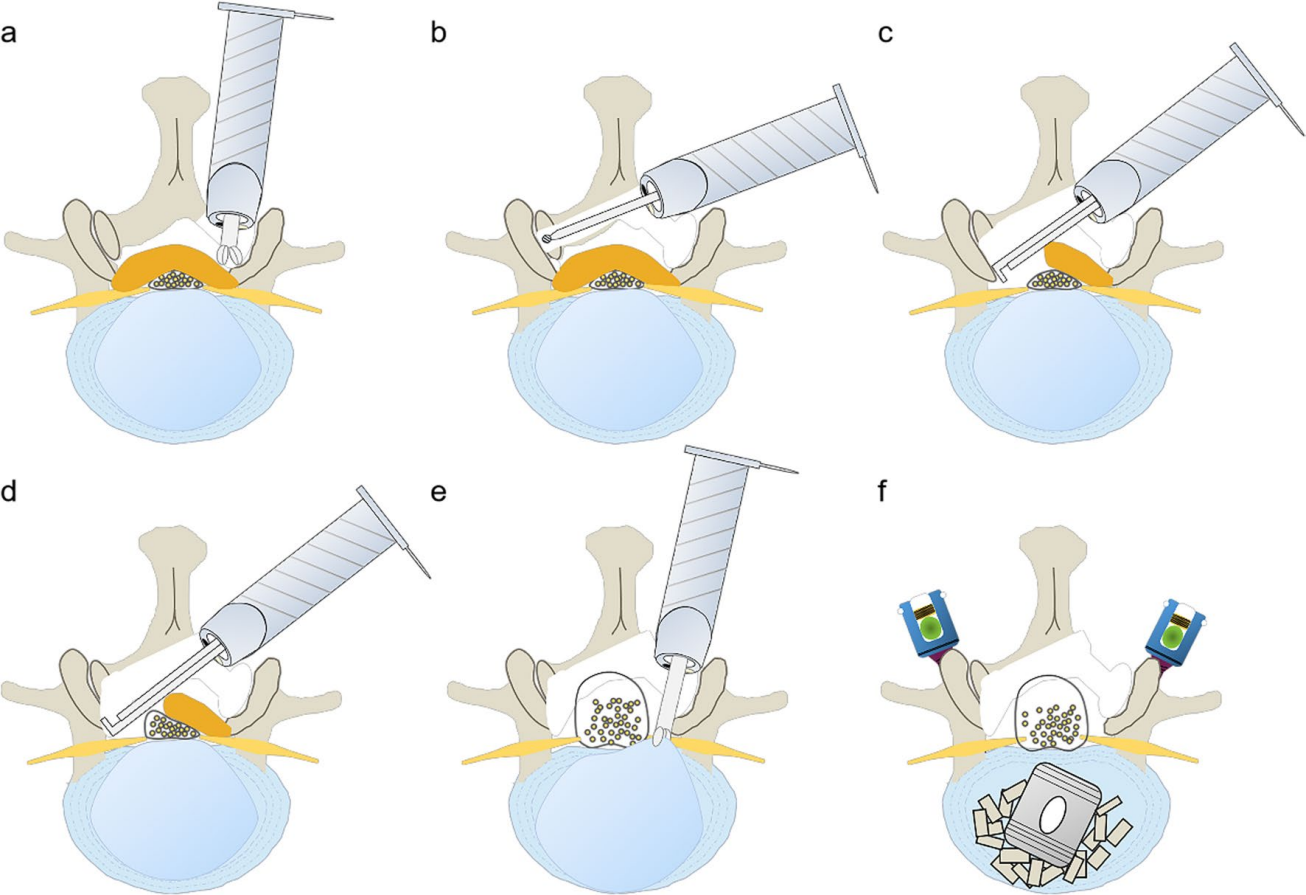
Six steps of the ‘Over-Top’ technique. **A** Ipsilateral laminectomy. **B** Decompression of the contralateral spinal canal while preserving the partial cortical bone near the LF.** C** Removal of the contralateral LF and the residual bone of lamina. **D** Decompression of contralateral LR. **E** Removal of the ipsilateral LF and discectomy.** F** Vertebral space bone grafting, cage and pedicle screws implantation. *LF* ligamentum flavum. *LF* ligamentum flavum, *LR* lateral recess

The key steps of the ‘Non-touch Over-Top’ technique are as follows. Firstly, the base of spinous process (BSP) is regarded as the anatomical marker of the starting point of performing ULBD, and the bony structure of BSP and the contralateral cranial lamina should be removed as much as possible. Decompression is performed by drilling between the laminae, with an effort to preserve the cortical bone near the LF as much as possible (Fig. 1). A sensation of sudden give is felt upon reaching the superior articular surface, after which this layer of cortical bone is removed. Next, the contralateral LF should be progressively removed using the endoscopic Kerrison rongeur (Fig. 2). Afterwards, the water pressure was reset at 100 mmHg. Through the combined effect of the irrigation water pressure and the dural sac’s tension, a spacious area is created between the dural sac and the contralateral lamina, and this space allows to observe and depress the contralateral LR easily. Meanwhile, through the collaborative effects of sturdy fixation of the pedicle screws and solid intervertebral fusion, spinal instability will not occur. Kim et al. [30] introduced specific twelve steps of ULBD under the traditional endoscope. Hua et al. [8, 9], whose study mentioned that preserving the LF would reduce damage and irritation to the dural sac and nerve roots during ULBD, claimed satisfactory clinical efficacy using the traditional small working channel endoscope when performing ULBD as well. However, we hold the perspective that the contralateral LF should be initially removed to expand the operative space and endoscopic sight during compressed with efficiency by large-channel endoscope.

**Fig. 3:**
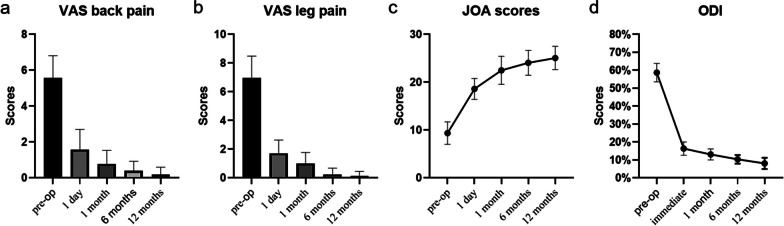
Visualization of clinical data of functional outcomes. Visual analogue scale (VAS) scores for back pain **a**, VAS scores for leg pain **b**, Oswestry disability index (ODI) scores **c**, and Japanese Orthopaedic Association (JOA) scores **d** showed a significantly improvement trend postoperatively compared with preoperative values

In this study, surgical efficiency and clinical efficacy has been significantly improved by the Non-touch Over-Top technique, which both ipsilateral and contralateral LR was completely decompressed simultaneously, 41 patients were followed for at least 6 months after surgery and none experienced spinal instability or internal fixation failure. The back VAS scores, leg pain VAS scores, ODI and JOA scores were recorded at four preoperative and postoperative time intervals to assess clinical efficacy. Back pain VAS decreased from 5.56 ± 0.20 to 0.20 ± 0.06, leg pain VAS scores decreased from 6.95 ± 0.24 to 0.12 ± 0.05, ODI decreased from 58.68 ± 0.80% to 8.10 ± 0.49%, and JOA scores increased from 9.37 ± 0.37 to 25.07 ± 0.38, which means these clinical indicators continued to improve significantly after twelve months of follow-up observation (*P* < 0.05) and the symptoms of the patients were significantly improved after surgery (Fig. 3). The fusion rate was 100% at the last follow-up, and the high fusion rate should be attributed to cleanly bone implanting beds (Fig. 1g).

The Cage subsidence rate was 2.44% (1/41) at both 6 months and 1 year postoperatively. It may be related to severe osteoporosis (T = − 4.7) in this patient [31, 32]. In addition, one patient suffered dural tear and postoperative cerebrospinal fluid leakage, and one patient suffered incision infection. Dural tear and CSF leakage occurred in the same patient during the early stages of applying this technique, likely due to improper use of the visualized trephine with excessive force.

**Fig. 4:**
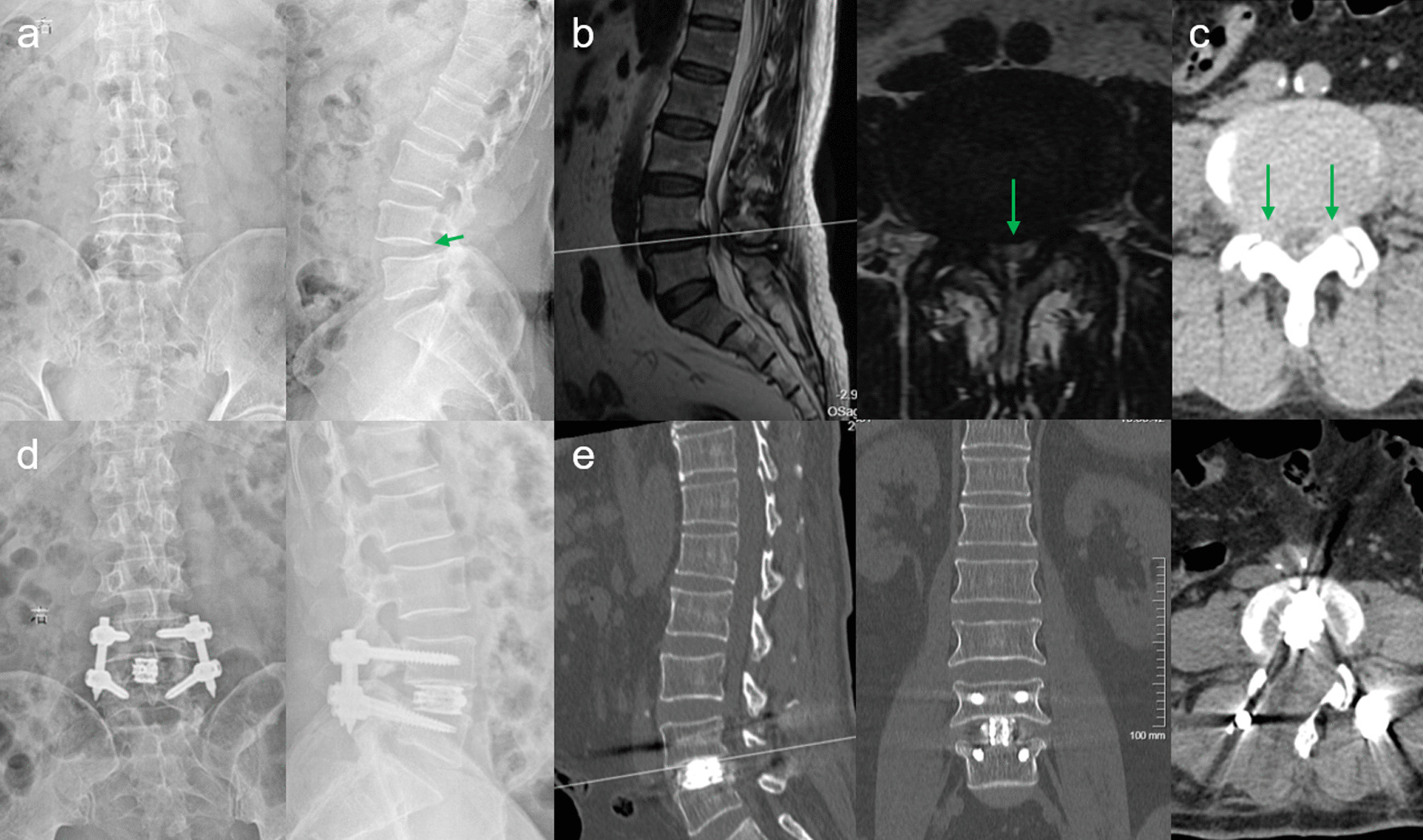
A 68-year-old male patient with severe intermittent claudication in both legs underwent PE-PLIF combined with the ‘Non-touch Over-Top’ technique. **a** preoperative AP X-ray fluoroscopy, and the green arrow showed I° spondylolisthesis at L_4-5_ segment. **b** preoperative MRI, the green arrow showed central canal stenosis at the L_4-5_ segment. **c** preoperative axial CT, and the green arrows showed bilateral LR stenosis at the L_4-5_ segment. **d** 1d postoperative AP X-ray fluoroscopy showed solid fixation of the implant and the successful reduction of the L_4-5_ segment. **e** 3d postoperative CT clearly demonstrated adequate spinal canal decompression. *PE-PLIF* percutaneous endoscopic posterior lumbar interbody fusion, *AP* anterior–posterior, *ULBD* unilateral laminotomy for bilateral decompression

Radiological examinations were performed before and after surgery to evaluate changes in various parameters. As shown in Table 4, LLA increased from 34.04 ± 0.25° to 45.56 ± 0.17°, SLA increased from 9.50 ± 0.11° to 20.71 ± 0.11°, and DH increased from 7.19 ± 0.13 mm to 9.15 ± 0.02 mm. This technique can effectively improve lumbar lordosis and intervertebral disc height. In addition, the results of axial CT examination showed that the CSAC increased from 98.20 ± 2.57 mm to 200.49 ± 4.73 mm, which was significantly improved compared with pre-operative results *(P* < 0.05, Fig. 4). The changes in CSAC indicate that PE-PLIF with ULBD can effectively improve the vertebral canal volume.

In conclusion, our findings suggest that the integration of PE-PLIF with the novel ‘Non-touch Over-Top’ ULBD technique via a large-channel endoscope is highly effective in the short-term management of LDD. It can not only significantly expand the spinal canal volume and fully decompress the nerve roots and dural sac, but also improve the lumbar sagittal parameters and stabilize the vertebral column. This novel technique markedly ameliorates the symptoms, boosting LSS patients’ quality of life and spinal stability. With minimal complications observed, this integrated technique emerges as a promising option for LDD treatment, warranting further exploration and optimization in future research.

### Supplementary Information

**Additional file 1.** Endoscopic view of decompressed contralateral spinal canal.

## References

[1] Bagley C (2019). Current concepts and recent advances in understanding and managing lumbar spine stenosis. F1000Res.

[2] Lurie J, Tomkins-Lane C (2016). Management of lumbar spinal stenosis. BMJ.

[3] Weinstein JN (2007). Surgical versus nonsurgical treatment for lumbar degenerative spondylolisthesis. N Engl J Med.

[4] Katz JN, Zimmerman ZE, Mass H, Makhni MC (2022). Diagnosis and management of lumbar spinal stenosis: a review. JAMA.

[5] Song Q (2021). Full-endoscopic lumbar decompression versus open decompression and fusion surgery for the lumbar spinal stenosis: A 3-year follow-up study. J Pain Res.

[6] Simpson AK (2022). Spinal endoscopy: evidence, techniques, global trends, and future projections. Spine J.

[7] Zhao XB, Ma HJ, Geng B, Zhou HG, Xia YY (2021). Percutaneous endoscopic unilateral laminotomy and bilateral decompression for lumbar spinal stenosis. Orthop Surg.

[8] Hua W (2020). Comparison of lumbar endoscopic unilateral laminotomy bilateral decompression and minimally invasive surgery transforaminal lumbar interbody fusion for one-level lumbar spinal stenosis. BMC Musculoskelet Disord.

[9] Hua W (2020). Comparison of clinical outcomes following lumbar endoscopic unilateral laminotomy bilateral decompression and minimally invasive transforaminal lumbar interbody fusion for one-level lumbar spinal stenosis with degenerative spondylolisthesis. Front Surg.

[10] Vandenbroucke JP (2007). Strengthening the reporting of observational studies in epidemiology (STROBE): explanation and elaboration. Ann Intern Med.

[11] Lim KT, Meceda EJA, Park CK (2020). Inside-Out approach of lumbar endoscopic unilateral laminotomy for bilateral decompression: a detailed technical description. Rationale and Outcomes Neurospine.

[12] Li XY (2023). Efficacy of oblique lumbar interbody fusion versus transforaminal lumbar interbody fusion in the treatment of lumbar degenerative diseases: a systematic review and meta-analysis. Arch Orthop Trauma Surg.

[13] Daniels AH (2016). Hospital charges associated with "never events": comparison of anterior cervical discectomy and fusion, posterior lumbar interbody fusion, and lumbar laminectomy to total joint arthroplasty. J Neurosurg-Spine.

[14] Ahn Y (2019). Endoscopic spine discectomy: indications and outcomes. Int Orthop.

[15] Sivakanthan S, Hasan S, Hofstetter C (2020). Full-endoscopic lumbar discectomy. Neurosurg Clin N Am.

[16] Kim HS, Wu PH, Jang IT (2020). Current and future of endoscopic spine surgery: What are the common procedures we have now and what lies ahead?. World Neurosurg.

[17] Kostysyn R (2023). Efficiency of interlaminar uniportal endoscopic lumbar discectomy. Bratisl Med J.

[18] Jiang C (2021). Full-endoscopic posterior lumbar interbody fusion with epidural anesthesia: technical note and initial clinical experience with one-year follow-up. J Pain Res.

[19] Wei FL (2021). Management for lumbar spinal stenosis: A network meta-analysis and systematic review. Int J Surg.

[20] Zhang J (2021). Decompression using minimally invasive surgery for lumbar spinal stenosis associated with degenerative spondylolisthesis: a review. Pain Ther.

[21] Ao S (2020). Comparison of Preliminary clinical outcomes between percutaneous endoscopic and minimally invasive transforaminal lumbar interbody fusion for lumbar degenerative diseases in a tertiary hospital: is percutaneous endoscopic procedure superior to MIS-TLIF? A prospective cohort study. Int J Surg.

[22] He LM (2022). Comparison of percutaneous endoscopic and open posterior lumbar interbody fusion for the treatment of single-segmental lumbar degenerative diseases. BMC Musculoskelet Disord.

[23] Wu J (2018). Percutaneous endoscopic lumbar interbody fusion: technical note and preliminary clinical experience with 2-year follow-up. Biomed Res Int.

[24] Heo DH, Lee DC, Kim HS, Park CK, Chung H (2021). Clinical results and complications of endoscopic lumbar interbody fusion for lumbar degenerative disease: a meta-analysis. World Neurosurg.

[25] Yoshikane K, Kikuchi K, Okazaki K (2023). Clinical outcomes of selective single-level lumbar endoscopic unilateral laminotomy for bilateral decompression of multilevel lumbar spinal stenosis and risk factors of reoperation. Global Spine J.

[26] Wu MH (2021). Outcome analysis of lumbar endoscopic unilateral laminotomy for bilateral decompression in patients with degenerative lumbar central canal stenosis. Spine J.

[27] Yoshikane K, Kikuchi K, Okazaki K (2021). Lumbar endoscopic unilateral laminotomy for bilateral decompression for lumbar spinal stenosis provides comparable clinical outcomes in patients with and without degenerative spondylolisthesis. World Neurosurg.

[28] Hua W (2022). Clinical outcomes of uniportal and biportal lumbar endoscopic unilateral laminotomy for bilateral decompression in patients with lumbar spinal stenosis: a retrospective pair-matched case-control study. World Neurosurg.

[29] Tan B, Yang QY, Fan B, Xiong C (2023). Decompression via unilateral biportal endoscopy for severe degenerative lumbar spinal stenosis: a comparative study with decompression via open discectomy. Front Neurol.

[30] Kim HS, Wu PH, Jang IT (2020). Lumbar endoscopic unilateral laminotomy for bilateral decompression outside-in approach: a proctorship guideline with 12 steps of effectiveness and safety. Neurospine.

[31] Yao YC (2020). Risk factors of cage subsidence in patients received minimally invasive transforaminal lumbar interbody fusion. Spine Phila Pa 1976.

[32] Amorim-Barbosa T (2022). Risk factors for cage subsidence and clinical outcomes after transforaminal and posterior lumbar interbody fusion. Eur J Orthop Surg Traumatol.

